# Teeth of Nations

**Published:** 1856-06

**Authors:** 


### TEETH OF NATIONS.

### INTRODUCTION.

Dear Doctor:

It is now over a year since I told you that I had taken notes on the condition of the teeth, among the different nations where I had traveled, and that I intended to furnish you, some day, with a series of articles on that subject, for the "Register," trusting that some of the facts might divert your readers, when their idle hours, which I fear are not uncommon, incline them to peer through their windows in search of custom. But the unsettled life I have been living, together with a portion of my time being given to writing light literature, too light, I fear, has caused me to defer it from time to time, in hopes that I could prepare my language with such technical phrases and incomprehensible meaning as might enable me to convince dentists that I had at one period of life succeeded in collecting a good deal of medical information. But the truth of it is, Doctor, I have become so habituated to "slicking" things over in my own style, that my laziness overcomes my ambition to gain renown, for the mysterious manner of expressing my thoughts; especially, too when I know so many learned eyes glancing over my words might detect the errors, into which any lofty attempt might plunge me. Ever since I told you about my notes, you have frequently teased me for a slice, as I am inclined to think, to lessen the amount of editorial matter, rather than to hope that you could gain any information from such a source; but so far, I have succeeded in putting you off, I believe, without your ever suspecting that my incapacity was my real internal apology. Though your solicitations have been quite often enough, and least you take offence and ask me no more, I see no escape but to sit down in the midst of my still unsettled life, and give you such a dose as may keep you quiet until I can get leisure to write with that stately deportment the "Register" requires.

But my subject is so wide, Doctor, and scattered among so many different people, that I scarcely know how to make selections, from my own notes, so as not to get my descriptions confused. To represent the general physiology of a nation, as a preliminary, seems almost indipensable, yet this is of no more consequence than to treat of the food and habits of the people; for the variations in the structure and health of the teeth, seem almost universally to conform to the state and condition of both of these; though in making such a remark, I do not mean that it shall be applied to individual cases, but to nations, in general That there are plenty of our own countrymen, whose teeth are very unhealthy, and whose systems are otherwise entirely free from desease, is certainly an indisputable fact; but that our nation is noted for defective teeth, and that their bony system is defective or inferior in strength and development to that of Europeans, is a fact that has long been known to all physiologists Again, were I to bring the aboriginees of Australia into comparison with our own people, their inferior bony structure, and poor teeth, I could not but come to the conclusion that their place and mode of living were still less adapted to the development of human beings than ours. But were I to bring the Scotch in comparison, noting that most of them are ignorant of diseased teeth, I could not pass unobserved their broad sholders and powerful limbs. Then there are other things that seem indispensible in the course of these remarks, for instance, arsenic and copper, exist in but small quantities in Scotland and England, but in Australia, fences are built of copper ore, and all the water, in most of the mineral districts is highly impregnated with arsenic, and no doubt many of the vegetables absorb large portions of it, and that through this medium it may assist in breaking down the human system; and in evidence that such sources act on the teeth is illustrated in the fact that petrifaction (salivary calculus) exists in more abundance in settlements where "iron water" abounds. But even in regard to the manner in which mineral substances produce these effects, there is such a field for investigation that we are too apt to neglect entering into the philosophy of the *modus operandi* by which the human system is nourished, and built up out of the substances that constitute the elements of food, but change our reflections too much to the chemical effect that such materials have on the external parts of the organs in question. Nearly all tooth philosophers commence illustrating these effects by chemical tests, in glass bottles, without ever dwelling on the principle of nourishment as being the cause of predisposition, notwithstanding, however, were they to hear a farmer say that good corn would grow on poor soil as well as rich, by bathing its temples, they would pronounce him mentally deficient.

The climate is also to be considered, not however, in the light that certain temperatures produce injurious effects on the teeth by the expansion and contraction of their surfaces, but rather through the medium by which closely knit, or languid systems derive their stamina from surrounding atmosphere. This must appear from the *ballancing tendency* of the system during the whole period of life, which undoubtedly produces its greatest influence during the development of the different organs. By *ballancing tendency*, Doctor, I do not exactly mean *sympathy*, but rather, that when *bracing atmosphere* or nutrition is deficient, that every organ is necessarily deprived of an equal share in the misfortune. And before I finish speaking of the comparative structure of the teeth of nations, of the physiology of certain climates, and of the teeth of people of different temperaments, I think, I will be able to clear up my views, also in reference to the deplorable fact that decayed teeth are more common in this, than in any previous age.

So you see, Doctor, aside from the mode of expression, I shall have quite as much to write as your readers will put up with; but should I get into any strange notions of theorizing for the sake of drawing out better talent from old dentists, you will do me a favor if you will give me a slight wink to withdraw before I am chased out of the field. Then, too, when I speak of some good dental operations, and some bad ones, that I have seen in this and other countries, you will please tell them that all persons who take the "Register" are not included in my remarks, when they bear heavily on buthering in general. But when I say a good deal in favor of American dentists, tell them not to be vain, for, there too, is talent abroad. And should the French and English epithets about 'snagled toothed Americans,' stir a spark of patriotism, point them to India, to the south of France, and to Italian lowlands, where they may gain inward satisfaction that Republicanism, and stump speeches did not cause the pains that others feel. Should the North condemn the South, tell them to keep their teeth; for fruit, talent and disease are joined together.

When I collected my notes I was many times troubled in gathering information, on account of not being able to speak the different languages, which was particularly the case among the North American Indians, and the aborigines of other countries. Among all of these, however, I was much surprised to find that many writers had been so negligent in detecting the condition of the teeth, so apt are they to call the contrast between a dark skin and white teeth, an indication of healthy ones, that they pen their impressions without examination. Then, again, in noticing the upper and lower classes of Europeans, good manners sometimes induced me to depend somewhat on the accounts that dentists gave me, rather than to muzzle every curious mouth I chanced across, for the sake of personal examination, which has, however, been the universal method I pursued when among such people as would permit of so friendly a liberty. I had intended, when first penning my observations, to give an account of the proportion of diseased teeth among different people, but the labor of collecting so much information induced me to abandon the idea; and in that respect I shall endeavor merely to form an estimate from my notes, as my judgment may dictate. At another period I had concluded to classify the food habits, climate, etc., so as to show the apparent effect of such on the teeth of nations comparatively considered, but this would have formed such a geometrical series as I never could have completed, so that, I shall now speak of each nation separately, or comparatively, as seems at the time most convenient for my own peculiar mode of writing.

Now, Doctor, if this letter pleases you, its publication as an introduction to a series of articles on this subject, will be a hint to me that I shall be at liberty to annoy your readers with my tiresome stories about other folks' teeth.

